# Supplementary material for: Three-dimensional experiments and individual based simulations show that cell proliferation drives melanoma nest formation in human skin tissue
Source: BMC Syst Biol. 2018 Mar 27;12:34. doi: 10.1186/s12918-018-0559-9 (PMC5872522; doi:10.1186/s12918-018-0559-9)
Supplement: Supplementary file 1 — Additional experimental results. (DOCX 2144 kb) [file 12918_2018_559_MOESM1_ESM.docx]

Three-dimensional experiments and individual based simulations show that cell proliferation drives melanoma nest formation in human skin tissue

Additional file 1

Parvathi Haridas^1, 2^, Alexander P. Browning^2^, Jacqui A. McGovern^1^, D. L. Sean McElwain^1, 2^, Matthew J. Simpson^1, 2^*

^1^ Institute of Health and Biomedical Innovation, Queensland University of Technology (QUT), Kelvin Grove 4059, Australia

^2^ School of Mathematical Sciences, QUT, Brisbane 4001, Australia

* [matthew.simpson@qut.edu.au](mailto:matthew.simpson@qut.edu.au)

Table of Contents

[1. Introduction 3](#_Toc495660019)

[2. Methods 3](#_Toc495660020)

[3. Results 3](#_Toc495660021)

[4. Conclusion 9](#_Toc495660022)

# 1. Introduction

This document provides additional information to support the results and discussion in the main manuscript. Here, we describe the experimental protocols in detail. We follow these protocols to obtain the results discussed in the main manuscript. Further, we disclose results from additional experiments. These outcomes supplement the overall findings documented in the main manuscript.

# 2. Methods

*2.1 Live cell assay*

We perform a live cell assay to study the morphological and behavioural differences between irradiated and non-irradiated melanoma cells. Melanoma cell suspensions of 5000/ml and 10000^­^/ml, are cultured using growth medium in wells of a 24-well tissue culture plate. The diameter of each well is 15.6 mm. The tissue culture plate containing cells is placed on the stage of a live cell imaging microscope (Leica DMi8, Australia) housed within an incubation chamber at 37 °C, in 5% CO_2_ and 95% air. Cells are monitored over 24 hours, and images are captured at t=0, 12 and 24 hours.

*2.2 Establishing 3D experimental skin model with melanoma cells and control skin model without melanoma cells*

We construct 3D experimental skin models following protocols in the main manuscript. We also construct control 3D experimental skin models following the same protocol except that melanoma cells are omitted.

# 3. Results

3.1 Confirmation that irradiated melanoma cells do not proliferate

The melanoma cells are gamma-irradiated to inhibit mitosis. We perform a live assay to provide evidence that irradiation does not change adherence or morphology, and to confirm that irradiated melanoma cells do not proliferate. Two initial densities of melanoma cells are used in this experiment. The assay is initialised with either 5000 or 10000 irradiated melanoma cells/0.5 ml growth medium, in a 24-well tissue culture plate. Equivalent experiments with non-irradiated melanoma cells are performed in triplicate. The assay is monitored for 24 hours in optimal growth conditions. Images are captured at t=0, 12 and 24 hours. The images in Figure S1A-F show that the irradiated melanoma cells have no change in adherence or morphology when compared to non-irradiated melanoma cells.

To confirm that irradiation prevents mitosis we count the total number of cells in each field of view, as a function of time, in the three experimental replicates. Figure S1G shows that non-irradiated melanoma cells proliferate as we see an increase in average cell number over 24 hours. However, the average cell number for experiments with irradiated melanoma cells remains constant over 24 hours (Figure S1H) confirming that the irradiated melanoma cells do not proliferate. Furthermore, the fact that the average number of irradiated melanoma cells remains constant with time suggests that there is no cell death. This shows that the melanoma cells survive gamma-irradiation.


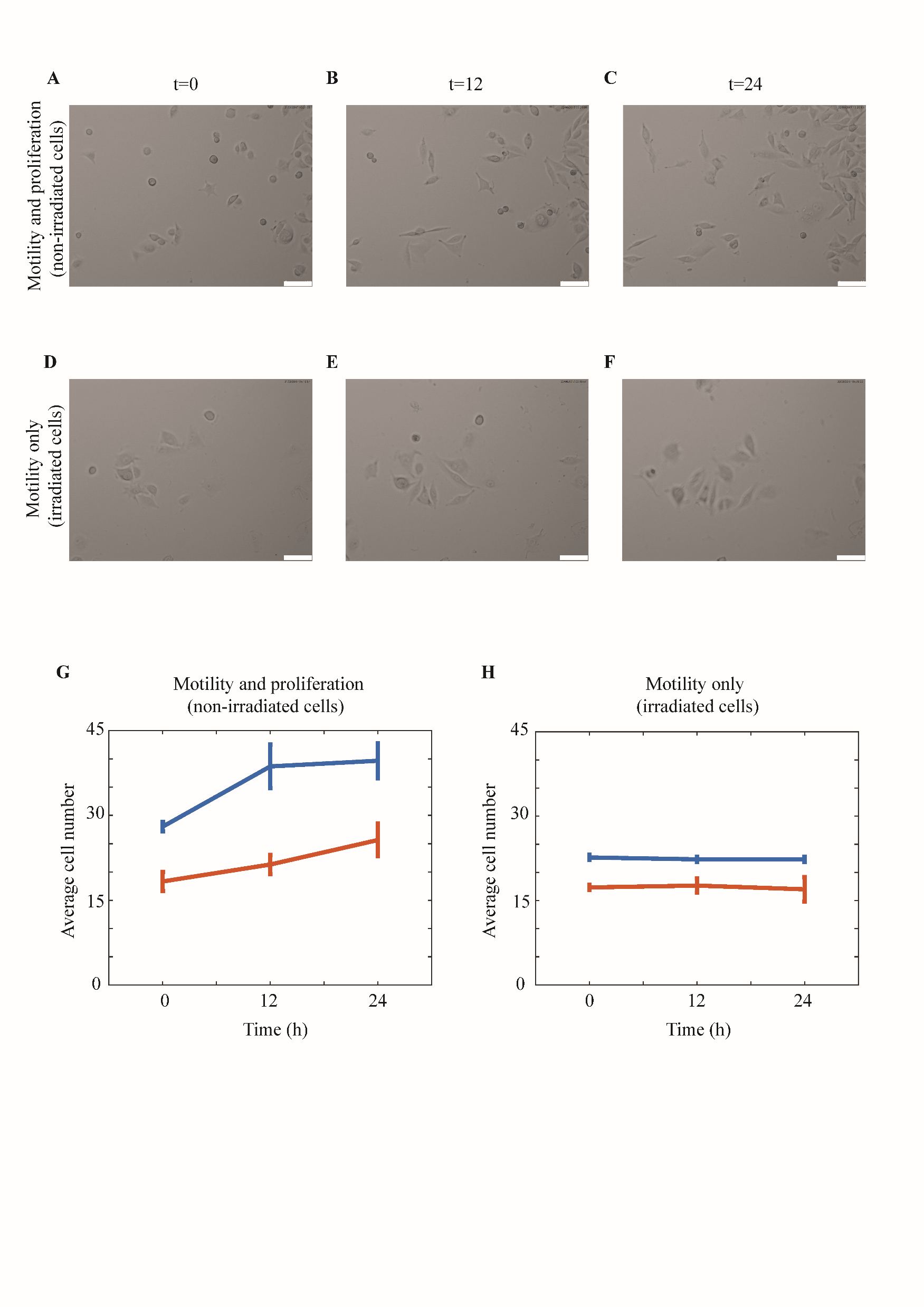


Figure S1: **Live cell assay of non-irradiated and irradiated melanoma cells.** Melanoma cells are cultured in a 24-well tissue culture plate, at approximately 10000 cells/0.5 ml growth medium. Sub-regions of size 0.92 mm × 0.69 mm are imaged. The images show: (A*-*C) non-irradiated melanoma cells; and (D-F) irradiated melanoma cells. The images correspond to t=0, 12 and 24 hours, respectively. The scale bar in each image is 100 µm. Plots show the average cell number (n=3) for: (G) non-irradiated melanoma cells; and (H) irradiated melanoma cells, as a function of time. We count the total number of cells in each field of view, as a function of time, in the three experimental replicates to calculate the average cell number. In both (G) and (H), the data points corresponding to experiments initialised with 10000 melanoma cells is in blue, and experiments initialised with 5000 melanoma cells is in red, respectively.

3.2 Absence of nests on control 3D experimental skin models

To provide evidence that nests are composed of melanoma cells on the 3D experimental skin model, we perform a set of control experiments. We construct control 3D experimental skin models in exactly the same way as the main experiments except that we omit melanoma cells. Therefore, the only cells present in the control experiments are the primary skin cells. The metabolic activity of all cells on the control 3D experimental skin models is observed using an MTT assay. Results for the control experiments are compared to the MTT assays performed on 3D skin models initialised with non-irradiated melanoma cells. In this case, we use a 3D skin model initialised with 5000 non-irradiated melanoma cells. The MTT assay highlights the metabolic activity of all cells on the surface of both 3D experimental skin models in purple (Figure S2). Results in Figure S2A show a complete absence of nests, while Figure S2B shows clear dark purple nests. This suggests that the dark purple staining on the 3D experimental skin model initialised with melanoma cells are clusters of melanoma cells.


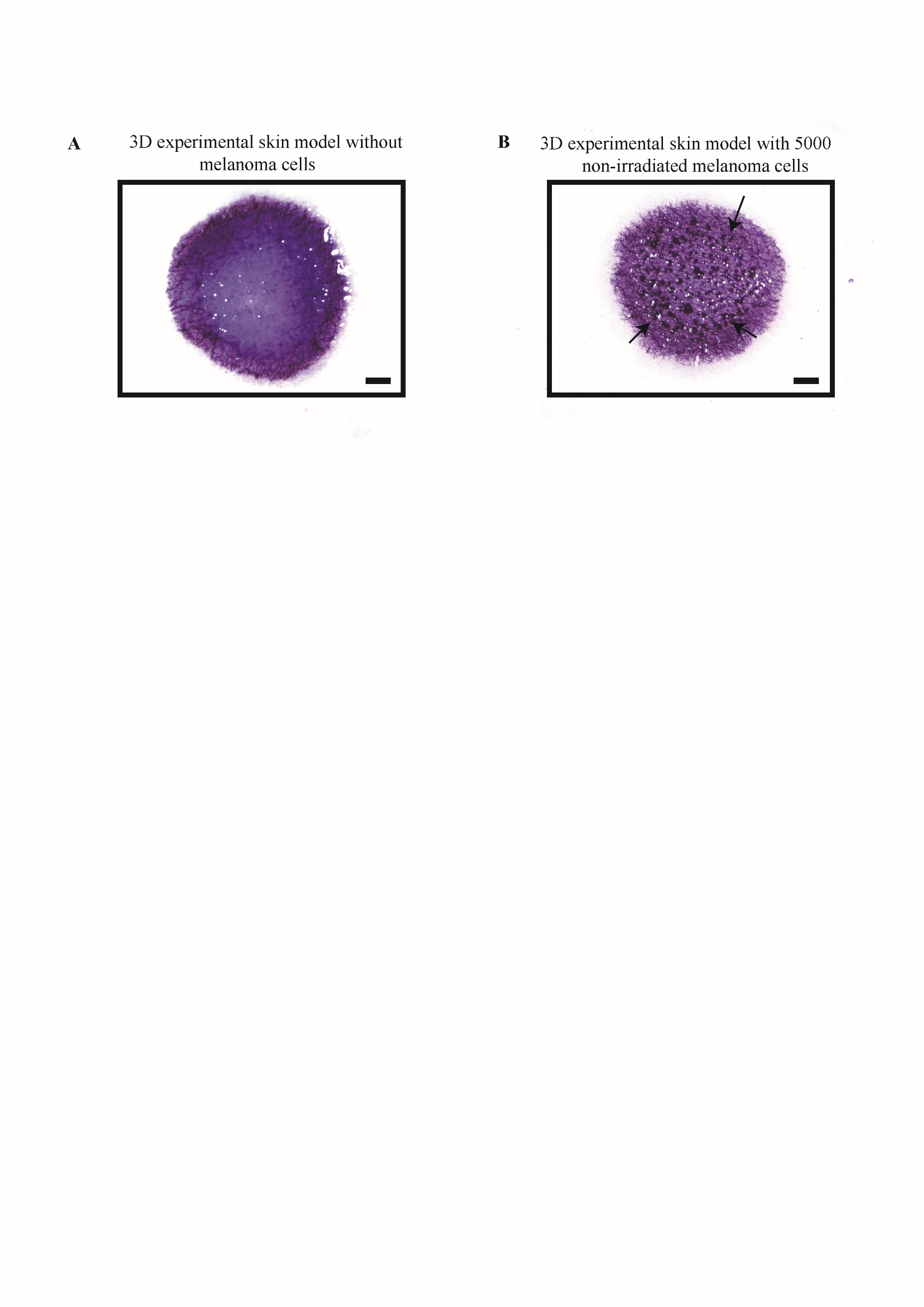


Figure S2: **Nest formation is absent on the 3D skin model without melanoma cells.** Images show metabolically active cells (light purple) on: (A) 3D experimental skin model without melanoma cells; and (B) 3D experimental skin model initialised with 5000 irradiated melanoma cells. Nests (dark purple) in (B) are indicated (arrows). The scale bar in each image is 1 mm.

# 4. Conclusion

These additional results confirm that irradiation does not alter melanoma cell morphology, adherence to substrate or their ability to migrate. Further, these additional results confirm that the dark purple nests on the 3D experimental skin model are clusters of melanoma cells.
